# Supplementary material for: The Effects of Electrolytic Technology Toothbrush Application on the Clinical Parameters and Bacteria Associated with Periodontal Disease in Dogs
Source: Animals (Basel). 2024 Oct 24;14(21):3067. doi: 10.3390/ani14213067 (PMC11544909; doi:10.3390/ani14213067)
Supplement: Supplementary file 1 [file animals-14-03067-s001.zip › animals-3175275-supplementary.pdf]

Supplementary Table S1. Dog breeds involved in the study

**Dog breed**	**No. of dogs**
Border Collie	1
Yorkshire Terrier	2
Pomeranian	1
Toy Poodle	3
Miniature Poodle	6
English Bulldog	1
Maltese Poodle	2
Bichon Frisé	3
Coton De Tulear	1
West Highland White Terrier	1
Mix breed / Without owner disclosure	5
